# Effective Pre-Training Method and Its Compositional Intelligence for Image Captioning

**DOI:** 10.3390/s22093433

**Published:** 2022-04-30

**Authors:** Won-Hyuk Choi, Yong-Suk Choi

**Affiliations:** Artificial Intelligence Laboratory, Hanyang University, Seoul 04763, Korea; gandet09@hanyang.ac.kr

**Keywords:** compositional intelligence, image captioning, feature mapping layer, pre-training method, transfer learning, transformer

## Abstract

With the increase in the performance of deep learning models, the model parameter has increased exponentially. An increase in model parameters leads to an increase in computation and training time, i.e., an increase in training cost. To reduce the training cost, we propose Compositional Intelligence (CI). This is a reuse method that combines pre-trained models for different tasks. Since the CI uses a well-trained model, good performance and small training cost can be expected in the target task. We applied the CI to the Image Captioning task. Compared to using a trained feature extractor, the caption generator is usually trained from scratch. On the other hand, we pre-trained the Transformer model as a caption generator and applied CI, i.e., we used a pre-trained feature extractor and a pre-trained caption generator. To compare the training cost of the From Scratch model and the CI model, early stopping was applied during fine-tuning of the image captioning task. On the MS-COCO dataset, the vanilla image captioning model reduced training cost by 13.8% and improved performance by up to 3.2%, and the Object Relation Transformer model reduced training cost by 21.3%.

## 1. Introduction

Recently, the parameters of deep learning models have increased exponentially. The parameter of GPT-3 [1] has a very large size of 175B. This is more than 500 times larger than the BERT large [2] model. As a result, the training cost increased significantly. In the case of GPT-3, the training cost of 3.14 × 10^23^ flops is almost 590 times that of the BERT large model. This is hundreds of years of work with a single NVIDIA V100 GPU and it costs over $4.6 million. Training these large models every time is expensive. Therefore, research to reduce the training cost is needed.

A common way to reduce training costs is transfer learning using pre-trained models. Transfer learning is solving one problem and then applying it to a different but related problem. In computer vision, a pre-trained feature extractor such as a VGG network [3] is used to fine-tune the target tasks [4,5,6,7]. In NLP, pre-trained BERT [2] is used to fine-tune the target tasks [8,9,10]. However, the previous methods take a pre-trained network and use it as part of the entire model, e.g., connect the pre-trained feature extractor and Region Proposal Network to detect objects in the image [5], and connect the pooling layer and softmax calssifier to the pre-trained BERT to perform sentence embedding [8]. So, part of the model is still training from scratch. The more parts to train from scratch, the higher the training cost.

The Compositional Intelligence (CI) method, on the other hand, constructs the entire model by combining pre-trained models. The advantage of using pre-trained models is that training converges quickly and performance can be improved. A representative model to which CI can be applied is the encoder–decoder structure. Since the encoder and decoder are connected with a hidden layer in the middle, they can be connected if the shape of the hidden vector is the same. It can be applied to various target tasks by combining a pre-trained encoder–decoder. Previous studies of CI have been done to combine two pre-trained models in the same domain. In the study by Yoo et al. [11], the image style transfer task was performed through a combination of encoder and decoder, trained with different style images. Oh et al. [12] applied CI to machine translation. First, the Transformer is pre-trained with monolingual task, and then the machine translation task is trained by connecting an encoder and decoder trained in different languages. Yoo et al. [11] applied the CI to the image domain and Oh et al. [12] applied the CI to the text domain. Since the same domain data is used, there is no need to consider the problem of domain differences when using CI. We extended CI to a dual domain; image and text. Two models trained in each domain were used for the image captioning task.

**Figure 1:**
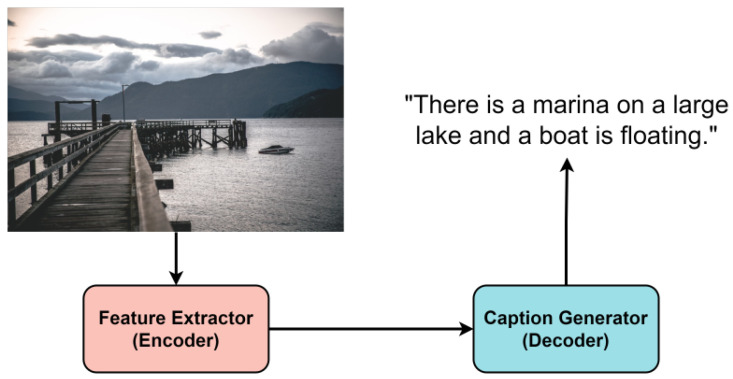
Typical image caption architecture. It consists of image feature extractor (encoder) and caption generator (decoder).

As shown in Figure 1, the typical image captioning model has an encoder–decoder structure. Most encoders use pre-trained feature extractors [3,5,13]. In the decoder, NLP models such as LSTM [14,15] and Transformer [16] are used as caption generators. After the announcement of the Transformer [16], which showed higher performance than the existing NLP model, a model using the Transformer as a caption generator was studied [17,18,19,20]. Since the model size of the Transformer [16] is much larger than that of LSTM [14], the training cost increased along with the performance improvement. Usually, the caption generators are trained from scratch, so training is expensive.

Since Image Captioning has an encoder–decoder structure, the CI method can be applied. Applying CI can reduce training costs while maintaining or improving performance. We used the pre-trained image feature extractor [21] commonly used in image captioning models. We devised and trained a proper pre-training task that works well as a caption generator. Connecting two pre-trained models on data from different domains requires feature mapping layer (FML) to mitigate differences in data distributions. We compared the training cost and metric scores [22,23,24,25,26] of our CI model that uses a pre-trained caption generator and the From Scratch model that trains the caption generator from scratch. For comparison of training cost, we checked the training time by applying early stopping when fine-tuning the image caption task. Compared to the From Scratch model, our CI model significantly shortened the training time, and all metric scores showed a slight improvement. In summary, our contributions of this study as follows.

- We extended our CI study from single domain to dual domain;
- We devised a pre-training task for caption generator;
- We showed in the image captioning task that the CI model is effective in reducing training costs.

## 2. Related Work

### 2.1. Image Captioning

Image recognition has come a long way in the ImageNet Large Scale Visual Recognition Challenge (ILSVRC) using ImageNet [27]. A CNN-based Deep Neural Network has been developed from AlexNet [28]. AlexNet won the ILSVRC-2012, significantly improving the top-5 test error rate of 26%, which was recorded by 2nd place, to 16%. Subsequent models, VGGNet [3], GoogLeNet [29], and ResNet [13], have better performance and deeper layers. The CNN network, which has been sufficiently trained in the challenge, has been used in several vision applications. Image captioning generates captions with visual information extracted through the CNN network. The CNN network fine-tuned VGGNet, GoogLeNet, and ResNet without training from scratch [30,31,32]. However, the aforementioned CNN network extracted one feature vector from the image. Since only one vector information was extracted from the image, the information entered into the caption generator was limited. With the advent of object detection models [33,34], it was possible to utilize the rich information by extracting object feature vectors from images [21]. The object detection model is used not only for image captioning but also for many vision applications [35,36,37]. All of the previous methods used a pre-trained CNN network, but all caption generators were used from scratch. Recently, models using Transformer [16] structure in image captioning are increasing [19,20,38]. If the Transformer structure is used, the parameters of the model inevitably increases, which leads to an increase in training cost.

### 2.2. Text Pre-Training Method

The field of NLP has been developed along with the study of various self-supervised pre-training methods. GPT-3 [1] trained using the decoder structure of Transformer [16]. It showed good performance with fine tuning in NLP tasks such as NLI, QA, reasoning, semantic similarity, classification, and text generation. The GPT-3 [1] also performed well for zero or few-shot learning, but it required a huge model size and a lot of data. BERT [2] uses the encoder of Transformer. BERT performed well with two objective functions: masked token prediction and next sentence prediction. However, BERT has poor text generation performance due to limitations in model structure and pre-training methods. Bart [39] has a standard Transformer architecture and trained the model using five pre-training methods: token masking, token deletion, sentence permutation, document rotation, and text infilling. These transformer models are expensive to train from scratch each time. Therefore, these models are fine-tuned to the target task using pre-trained models. We used the original Transformer [16] as a pre-training model so that both the encoder and decoder can be reused. We also devised a pre-training method to predict a sentence with keywords suitable for caption generator.

### 2.3. Compositional Intelligence Method (CI)

**Figure 2:**
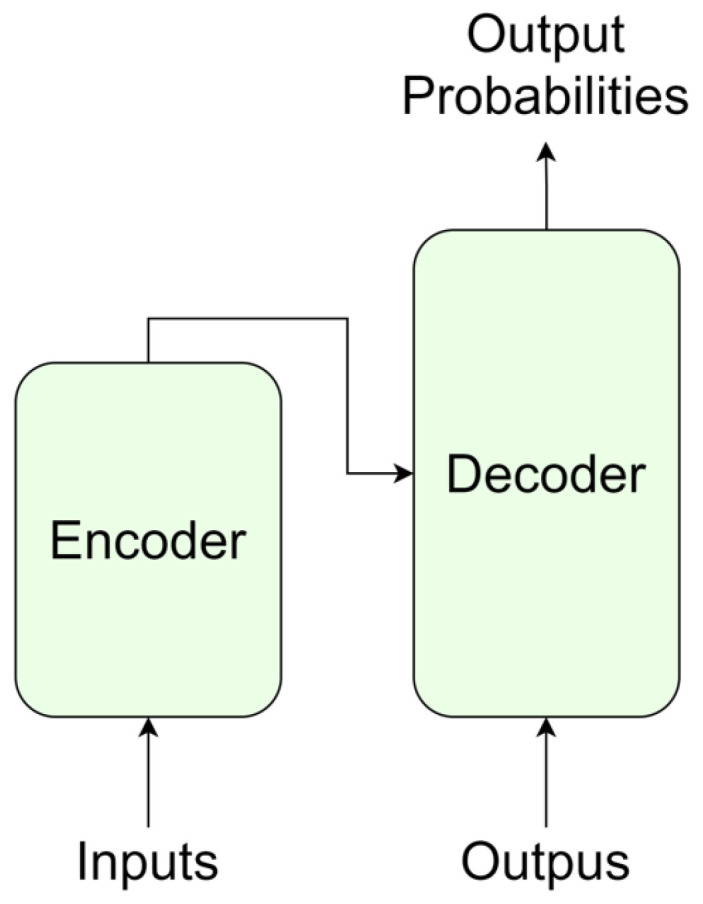
Original Transformer architecture. The Transformer consists of encoder and decoder structures.

CI is used to connect models learned in different domains or tasks and apply them to a new task. It is a type of transfer learning in that it uses pre-trained models. However, transfer learning can use a part of the model as a pre-trained model, but CI uses only pre-trained models except for a simple FML. CI utilizes a pre-trained model and can expect effective performance with minimal training cost. The model structure in which CI can be easily applied is the encoder–ecoder structure. Various combinations of models can be created with multiple encoders and decoders. Yoo et al. [11] performed well on style transfer tasks using Auto-encoder. In Auto-encoder learned with Style-A data, encoder extracts A style’s presentation, and decoder regenerates image from the presentation. If the encoder learned in A style and the decoder learned in B style are connected to the feature mapping layer, the image of Style A is converted into the image of Style B. Similar studies create one shared latent space of multiple style data, such as UNIT [40] and CD-GAN [41]. As shown in Figure 2, the Transformer has encoder and decoder structures. Therefore, CI can be applied by connecting the encoder trained with Data A and the decoder trained with Data B. Oh et al. [12] conducted a study on translating languages by combining multiple Transformers trained on monolingual data. We extended the CI method to dual domains. We applied CI to the image captioning task by reusing the feature extractor and caption generator.

## 3. Approach

A typical image captioning model uses a pre-trained image feature extractor, but a caption generator is trained from scratch. Unlike this, we pre-trained the caption generator and conducted image captioning training using CI. A pre-training method suitable for the target task to which CI is to be applied is required. Inappropriate pre-training methods can cause performance degradation during fine-tuning. Therefore, we devised and applied a pre-training method suitable for image caption generator.

### 3.1. Pre-Training Model Architecture

**Figure 3:**
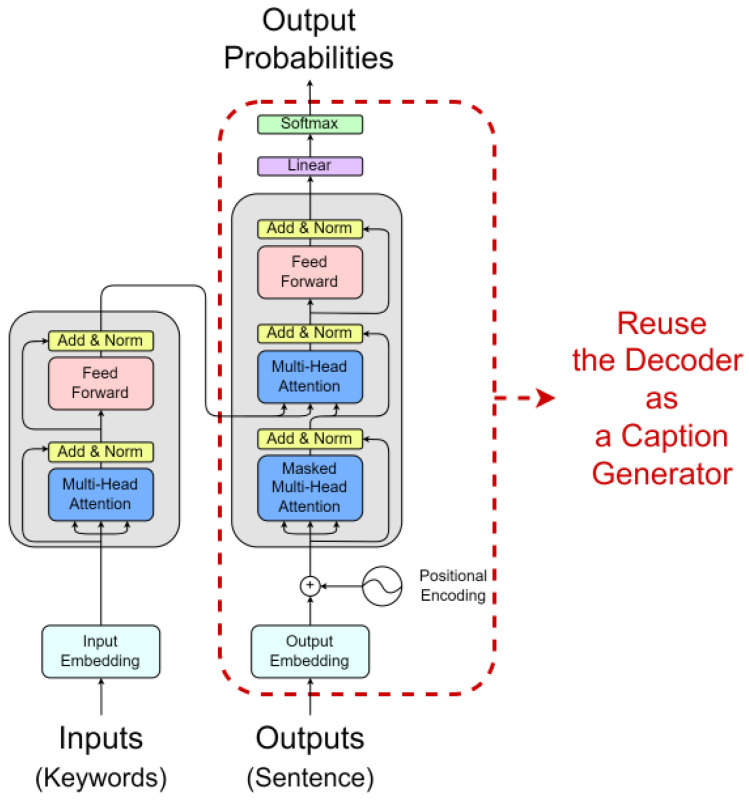
Pre-training model architecture.

**Figure 4:**
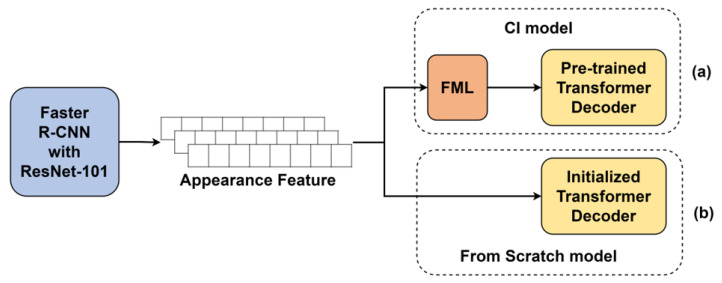
Two models used in the image captioning task. (**a**) Image captioning model using pre-trained caption generator. (**b**) Image captioning model with a caption generator trained from scratch.

We used the Transformer [16] base model as the pre-training model. As shown in Figure 2, the Transformer is divided into encoder and decoder structures, and the decoder is responsible for generating text. So, we reused the pre-trained decoder as a caption generator. We used keywords as input to the pre-training, but there is no need for positional information between the keywords. This is because, regardless of the order of keywords, the model completes the sentence by putting the keyword in the appropriate position when generating the sentence. Therefore, Positional Encoding (PE) used in the original Transformer encoder was removed. As shown in Figure 3, the pre-training model was constructed. We extract keywords from sentences and use them as input to the encoder. The decoder calculates the attention score between the keywords passed through the encoder and the tokens predicted previously ($Token_{0},Token_{1}\cdots Token_{t-1}$). The decoder updates the values of the tokens with the attention score and predicts the next token ($Token_{t}$). As shown in Figure 4, the CI model requires FML because of the different vector distributions of the two pre-trained models. On the other hand, the From Scratch model does not need FML because it trains the caption generator from scratch.

### 3.2. Pre-Training Method

It is impossible to unconditionally increase good performance by connecting pre-trained models to each other. Therefore, in order to harmoniously connect the models, it is necessary to devise a pre-training method suitable for the target task. We used two pre-trained models for the image captioning task. As shown in Figure 1, the image captioning model consists of two parts, the first part is the image feature extractor and the second part is the caption generator. As shown in Figure 4, Faster R-CNN composed of ResNet-101 was used as an image feature extractor [21]. Between 10 and 100 feature vectors of 2048-dimension are created for each image.

**Figure 5:**
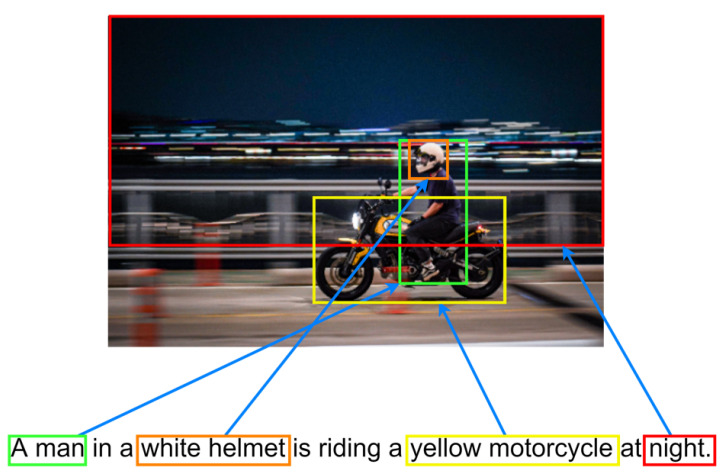
The relationship between the objects in the image and the keywords in the sentence.

Each object feature vector extracted from the image feature extractor is fed into the caption generator. That is, object information is used to create captions. The caption generator learns to insert verbs or adjectives into relationships between objects when there is no information other than objects. As shown in Figure 5, it can be seen that the object detected in the image and the keyword of the sentence play the same role. Therefore, we assumed that if the caption generator was pre-trained to construct a sentence with keywords, it would be suitable for working with the image captioning task.

**Figure 6:**
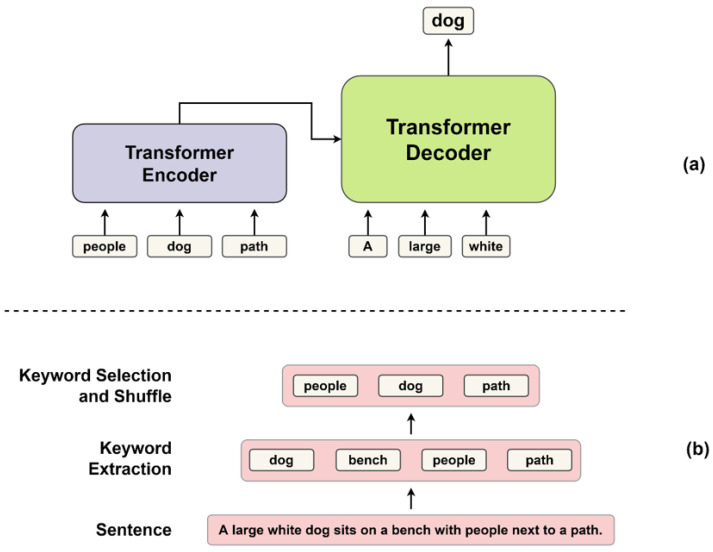
Input generation and training method in pre-training phase. (**a**) When keyword data is fed into a pre-training model encoder, the decoder learns to generate the original sentences. (**b**) Shows how to create input data from a sentence. After extracting keywords, we select some and then shuffle.

Figure 6 shows the training process and input of the pre-training model. The process of generating the input is divided into two parts, as shown in Figure 6b. First, we extract keywords from the sentence. Keywords were created by dividing sentences into word units and excluding stopwords [42]. Then, we select some of the keywords and shuffle to make them input data. Training was conducted using only 70% of the keywords made in one sentence. The use of 70% of keywords was determined through experiments, in Section 4.3.1. Table 1 is an example of the input actually made from the sentence. As shown in Figure 6a, the generated input is fed into the encoder of the pre-training model, and the decoder is trained to generate the original sentence. A word tokenizer was used instead of sub-words when tokenizing keywords to match object feature units in the image to be used for fine-tuning. We used the pre-trained Transformer-xl tokenizer [43] of Huggingface [44].

Since our pre-training method creates input keywords from a single sentence, an Input-Output data pair is not required. Therefore, it has the advantage of being able to easily obtain the data required for pre-training. We used WikiText-103 dataset [45] for pre-training. In order to have a distribution similar to the MS-COCO caption, which is an image captioning dataset, several preprocessing steps were performed. The maximum length of the MS-COCO caption was 250 and the minimum length was 21. Therefore, wiki sentences with a length of less than 21 characters or more than 250 characters were excluded. Pre-training was performed for 5 epochs with about 730,000 data.

### 3.3. Feature Mapping Layer (FML)

**Figure 7:**
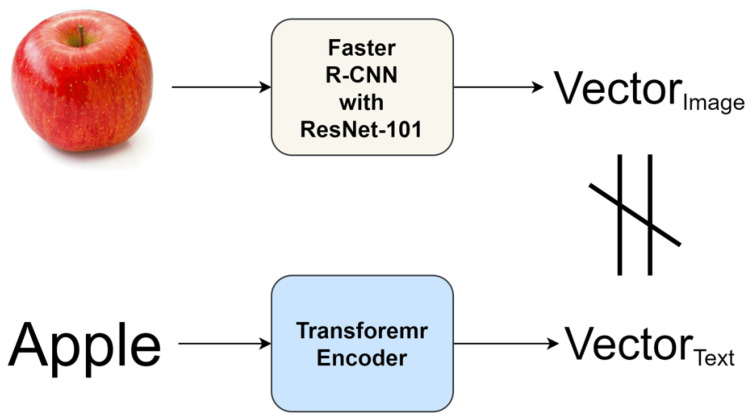
Compare vector values of images and words.

We pre-trained the model in the image domain and text domain. As shown in Figure 7, images and words of the same meaning trained in different domains have different vector values. This is because different models have different distributions of feature spaces they create. So we need a layer that smoothly maps the different feature spaces. Previous studies also showed that the model with FML outperformed the model without FML [11,12]. From the experimental results in Section 4.3.2, the Overcomplete FML showed the best performance. FML consists of two fully-connected layers. Using ReLU, a non-linear mapping function can be created.

### 3.4. Compositional Intelligence (CI)

As shown in Figure 4, a CI method was used to connect the pre-trained image feature extractor and caption generator with FML. We used a model composed of Faster R-CNN [5] and ResNet-101 [13] as a pre-trained image feature extractor. However, we did not train the model and used the extracted image vectors provided by Anderson et al. [21]. Anderson et al. pre-trained the image feature extractor with the Visual Genome dataset [46], and extracted feature vectors of objects from the images of the MS-COCO dataset [47]. The image of the bounding box created through the Region Proposal Network (RPN) of Faster R-CNN was given as input to ResNet-101. An intermediate feature map of ResNet-101 was used. For each bounding box, 2048-dimensional vector values were extracted.

Before proceeding with CI, we reduced the 2048-dimensional vector to 512-dimensional using an auto-encoder. The reason is that it was not possible to use a 2048-dimensional vector as it is in our experimental environment. Through the test, it was confirmed that there was no performance degradation due to dimensional reduction. The vector is input as the Key and Value of the second Multi-Head Attention of the Transformer decoder, which is a caption generator, through FML. The caption generator gets its weights from a Transformer model that has been pre-trained with keywords. The completed model has a structure in which Faster R-CNN with ResNet-101 and Transformer decoder are connected through FML. Then, fine-tuning of the image captioning task is performed on the MS-COCO dataset.

## 4. Experiment

### 4.1. Dataset and Metrics

For the image captioning task, we used the MS-COCO 2014 captions dataset [47]. Training and evaluation were conducted with Karpathy validation and test splits [48]. The splits divide the MS-COCO dataset, and as shown in Table 2, it consists of 113 K train data and 5 K each of validation and test data. Instead of training an image feature extractor, we used image feature vectors extracted by Anderson et al. [21] from the MS-COCO dataset. Anderson et al. provides between 10 and 100 2048-dimensional object feature vectors in an image. We mentioned in Section 3.4 that we reduced the 2048-dimensional feature vector to 512-dimensional. This is because we had an issue with the GPU memory in our experimental environment (we used one Nvidia GeForce RTX 2080 Ti). As model evaluation indicators, CIDEr-D [22], SPICE [23], BLEU [24], METEOR [25], and ROUGE-L [26] are used. It is common to evaluate image captioning models with these metric scores. To compare the training cost, the training time was measured by applying early stopping. There was a difference applying the CI, but other training environments (epochs, batch size, model hyperparameters, and hardware environment) were the same (to be exact, the parameters of the CI model are a little more because of the FML. However, the CI model overcame this weakness and showed better performance). Therefore, the shorter the training time, the lower the training cost.

### 4.2. Vanilla Image Captioning Model

As mentioned in Section 3.4, we experimented with two methods: training the image captioning model from scratch and applying CI to the pre-trained model. As shown in Figure 4, in the CI model, the image feature vector goes into the Transformer decoder through FML, and in the From Scratch model, the image feature vector goes directly into the Transformer decoder without FML. As a result of the experiments in Section 4.3.1 and Section 4.3.2, we used a pre-trained model with 70% keyword input and an Overcomplete FML in this experiment. We used the decoder of the Transformer base model as the caption generator. It has 6 decoder layers (*N*), 8 heads (*h*), 512 embedding vectors ($d_{model}$), and a size of 2048 feed-forward network ($d_{ff}$). The batch size is 20. We use Adam optimizer without the learning rate scheduler (Learning rate = $9\times 10^{-5}$, $\beta_{1}=0.9$, $\beta_{2}=0.999$ and $\epsilon =1\times 10^{-6}$). Except for FML, the training conditions of the CI and From Scratch models were the same. As shown in Table 3, the CI model has 2.1M more parameters than the From Scratch model due to FML. Both models were trained 8 times.

#### 4.2.1. Evaluation Metric Scores and Training Cost

The advantage of the CI model is that it can reduce the training cost by using a pre-trained model. Therefore, when training the CI and From Scratch models, we applied early stopping to measure the time the training ends. Since the training conditions were the same, the time spent on training equals the training cost (as can be seen in Table 3, although the CI model has 3.74% more parameters due to FML than the From Scratch model, but had a greater advantage in training time than that). Early stopping conditions are as follows:

$$L_{val}\left(t\right)>L_{val}\left(t-2\right),$$

where $L_{val}$ is the loss for the validation data after each epoch training and the *t* is the epoch. After each epoch, the validation loss was measured and the training was set to end if the loss was greater than before 2 epochs. For the CI model, it exhibits the lowest validation loss at 4 epochs, so it overfits at early stopping conditions greater than 2. When the model was continuously trained, both the CI model and the From Scratch model showed the lowest values around 4–6 epochs, and it was confirmed that they continued to increase.

**Figure 8:**
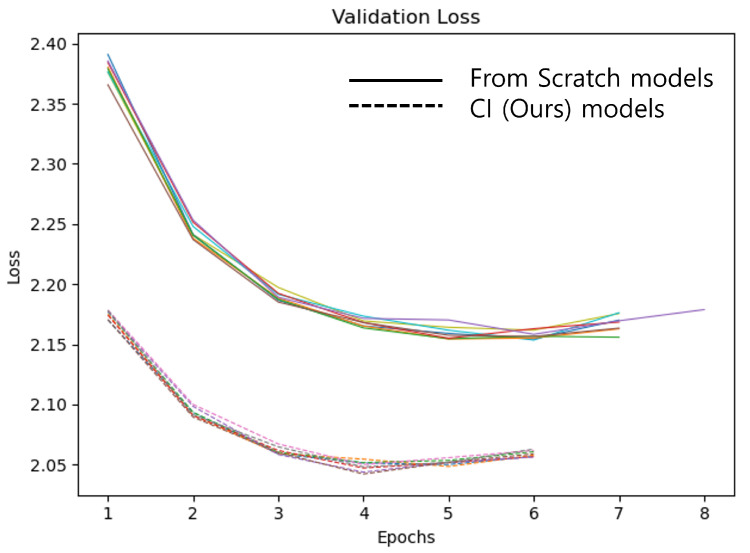
Vanilla image captioning model validation loss. The solid line are the From Scratch models and the dotted line are the CI models (ours). Each model was trained 8 times and is marked with different colors.

Figure 8 shows the measurement of validation loss of each model. Early stopping was applied to stop training when the current validation loss was higher than before 2 epochs. It can be seen that the tendency is similar, depending on the model. For the CI model, training was finished at 6 epochs during a total of 8 training sessions, and the From Scratch model was trained for 7 epochs and 8 epoch. As shown in Table 3, comparing the time used for actual training, the CI model saved about 13.8% of the time compared to the From Scratch model. It was confirmed that the CI model has the effect of reducing the training cost compared to the From Scratch model. Table 4 is the result of generating captions for the test dataset and measuring the metric scores. Mean and standard deviation metric scores were calculated for the model trained 8 times. We confirmed that the CI model has a small performance improvement in all metric scores compared to the From Scratch model. In particular, in the case of CIDEr-D, there was a performance improvement of 3.2%. Therefore, the CI model can significantly reduce the training cost while being similar or slightly better in the metric score.

#### 4.2.2. Qualitative Analysis

Table 5 shows the qualitative results of the From Scratch model and the CI model. We selected two images from the test dataset and performed inference (1 step = 1 batch). Looking at the inference results before training (0 step), both the From Scratch model and the CI model repeat the same word. In the case of the CI model, before the start of training, the image and text mapping is not done, so it generates words that have nothing to do with images, but unlike From Scratch, it tries to make a sentence. In the case of 200 steps, the From Scratch model still creates only the word ‘a’, but in the case of the CI model, as the mapping layer is learned, it starts to generate sentences similar to images. In the case of the From Scratch model, the sentence generation for the image above started at step 2600, and the sentence generation for the image below started at step 1600. As learning progresses, it can be seen that both the From Scratch and CI models generate sentences of similar quality. It can be confirmed that the CI model learns faster than the From Scratch model, but both models quickly generated sentences that fit the images. Since the captions of the MS-COCO dataset consist of relatively simple sentences, it can be considered that the learning is fast. If CI is applied to complex or long sentence data, better results can be expected.

### 4.3. Ablation Study

#### 4.3.1. Comparison Input Keyword Percentage

We pre-trained the models with different keyword input ratios. We thought that if 100% of the keyword extracted from the sentence is used, the pre-training model can easily predict the original sentence. If only 30% of the keyword is used, the original sentence cannot be easily predicted, but it creates a creative sentence. Therefore, a model with a low percentage of keywords is more effective when the input information is scarce. When fine-tuning the image captioning task, the information of image objects received as input may not be sufficient. Therefore, this experiment aimed to find the optimal pre-training model.

The training was carried out by setting the keyword input of the pre-training model to 30%, 50%, 70%, and 100%. As in Section 4.2, we applied CI to the image captioning task. We then evaluated which pre-trained model had the best metric scores. Each model was trained 8 times and the mean and standard deviation were calculated. As shown in Table 6, it was confirmed that the metric performance of the 70% model was good overall. When comparing the training epoch by applying early stopping, the 70% model and the 50% model showed good performance with an average of 6 epochs. We used the keyword 70% pre-training model to evaluate the vanilla image captioning models in Section 4.2 considering the metric score and the training cost.

#### 4.3.2. Comparison Feature Mapping Layer

**Figure 9:**
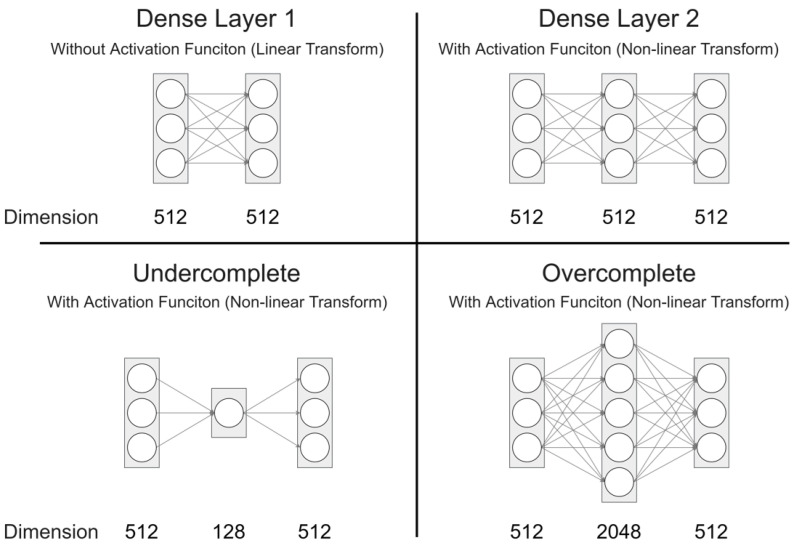
Four Feature Mapping Layers used in the experiments in Section 4.3.2.

In order to find an effective FML, we devised four FMLs, as shown in Figure 9. To see which performed best, we experimented with a model without FML and four FML models. The model without FML is a model to check whether FML is effective in our study as in the experiments of previous studies [11,12].

The without FML model has the same structure as the From Scratch model in Section 4.2, but the caption generator performed fine-tuning by loading the weights of the pre-trained model. For Dense Layer 1, the FML equation is

$$FML\left(x\right)=xW_{1}+b_{1},$$

where $W_{1}\in R^{512\times 512}$. Dense Layer 1 has a single dense layer, but the activation function is not applied. Therefore, it is an FML that acts as a linear transform. The other FML expressions are as follows:

$$FML\left(x\right)=max\left(0,\;xW_{1}+b_{1}\right)W_{2}+b_{2}.$$

For a Dense Layer 2, $W_{1}\in R^{512\times 512}$ and $W_{2}\in R^{512\times 512}$. It consists of two dense layers, and a non-linear transformation was performed by applying a ReLU activation function in the first layer. For a Undercomplete, $W_{1}\in R^{512\times 128}$ and $W_{2}\in R^{128\times 512}$. This structure is similar to the auto-encoder. For a Overcomplete, $W_{1}\in R^{512\times 2048}$ and $W_{2}\in R^{2048\times 512}$. It has the same structure as Transformer’s Feed-forward Block. The ReLU activation function was applied to the first layer for both Undercomplete and Overcomplete.

As shown in Table 7, early stopping was applied to each model, and evaluation was performed after training seven times to obtain the mean and standard deviation. The performance of the model using Overcomplete FML showed better performance in all metrics than other models. In addition, the training cost was lower compared to other models. The *w/o* FML model had a small training cost, but the metric scores were all lower than other models. In the case of Undercomplete, it was confirmed that the performance was the lowest and the training cost was large. It can be inferred that as the size of the vector in the intermediate layer becomes smaller, the information loss increases.

### 4.4. Apply CI to Object Relation Transformer

**Figure 10:**
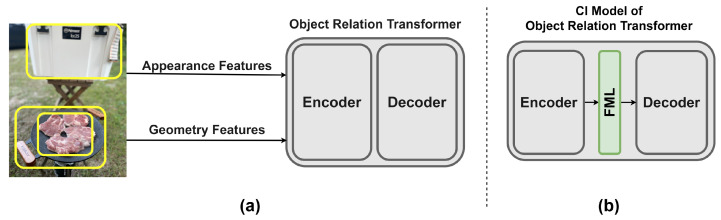
Object Relation Transformer (ORT) model architecture. (**a**) The ORT model devised a new encoder block to process image features and geometry features. (**b**) ORT model structure using FML to apply CI method. We used the Dense Layer 2 FML.

We experimented with whether CI was applicable to the model of the previous paper. We applied CI to Object Relation Transformer (ORT) [38]. It is an image captioning model using Transformer. As shown in Figure 10a, the ORT model generates captions after passing the image feature vectors and geometry features, which are the bounding box information, through the encoder. Unlike the vanilla image captioning model in Section 4.2, since the ORT model is a structure in which encoders exist, both the CI and the From Scratch models had to train the encoder from scratch. This is because the encoder architecture of our pre-training model is different from the encoder architecture of the ORT. Unlike the original Transformer encoder, the ORT encoder computes the relation between appearance features and geometry features.

The From Scratch model was trained using the ORT model as it is, as shown in Figure 10a. When learning the image captioning task, both the encoder and decoder of ORT were trained from the scratch. The CI model has a model structure in which FML is connected between the ORT encoder and the decoder, as shown in Figure 10b. The Dense Layer 2 FML mentioned in Figure 9 was used. Because the encoder of the ORT model has a different architecture from the pre-trained model encoder in Section 3.1, pre-trained weights could not be used. However, in the case of the decoder, since the model structure is the same, fine-tuning was performed using pre-trained weights (we used the weights of the 70% keyword pre-trained model). Therefore, in the case of the CI model, when fine-tuning, the encoder and FML were trained from scratch. Except for the structure of the model, other training conditions were equally applied to both the From Scratch model and the CI model. We used 6 encoder and decoder layers (*N*), 8 heads (*h*), 512 embedding vectors ($d_{model}$) and a size of 2048 feed-forward network ($d_{ff}$) for the ORT model. The batch size is 16. We use Adam optimizer without learning rate scheduler (Learning rate = $4\times 10^{-4}$, $\beta_{1}=0.9$, $\beta_{2}=0.999$ and $\epsilon =1\times 10^{-8}$). As shown in Table 8, the CI model has 0.6M more parameters than the From Scratch model due to FML. Each model was trained 7 times by applying early stopping, and the mean and standard deviation were measured by evaluating the metric scores.

As shown in Table 9, although the CI model has lower scores for all metrics compared to the From Scratch model, the value is around 1%, so it can be seen that there is no significant difference in performance. However, the training cost results are impressive. As shown in Table 8, the CI model was able to save 21.3% of the training time of the From Scratch model. ORT’s CI model is not the original CI method because the encoder must be trained from scratch. This is because the original CI approach was to fine-tune two pre-trained models by connecting them with a simple FML. For this reason, the metric scores may not have the same results shown in Table 4. However, there was an impressive performance improvement in the training cost. We mentioned that the training cost can be reduced with the CI method as the parameters of the model are increased. Compared to the vanilla image captioning model in Section 4.2, the parameters of the ORT model is about 5.5 times larger. The training cost save rate increased from 13.8% to 21.3%. If the pre-trained model was used even for the encoder of the ORT model, the performance would have been better not only in the metric scores but also in the training cost.

## 5. Conclusions

In this paper, we conducted a study by extending mono-domain CI [11,12] to dual-domain CI. CI uses pre-trained models to create a model for a new target task. We applied CI to the image captioning task by combining a model trained on images and a model trained on text. The previous image captioning models only use a pre-trained feature extractor and the caption generator is trained from scratch. We devised a pre-training method of caption generator that extracts keywords, puts them as input, and predicts original sentences. Since the objects of the image and the keywords of the sentence play the same role, it was effective when CI was applied to the image captioning task. We show that training the CI model is faster compared to the From Scratch model. The metrics maintained similar scores to the From Scratch model. That is, the CI model trained quickly and the performance was the same. The advantage of the CI method is that it uses the pre-training model multiple times. We applied CI to the vanilla image captioning model in Section 4.2 and the ORT model in Section 4.4 with the same pre-trained captiong generator. The more the pre-trained model is used for multiple target tasks, the more the cost can be reduced. As a future study, we will conduct research by expanding the domain to voice-to-text, video-to-text, and text-to-image. After that, the study will be expanded from dual-domain to multi-domain. As the name suggests, we will conduct research for Compositional Intelligence.

## Figures and Tables

**Table 1:** Input and output for the pre-training phase. Keywords are extracted from a sentence.

Input (Keywords)	Output (Sentence)
[Democrats, without, 1920, Beckham]	The Democrats renominated Beckham without opposition in 1920.
[Route, 1, interchange, northbound, entrance]	Route 1 at a partial interchange with a northbound exit and southbound entrance.
[Two, 1963, 1964, rerouted, western, highway]	Two realignments in 1963 and 1964 rerouted the western end of the highway again.

**Table 2:** Statistics of MS-COCO 2014 dataset.

Train	Valid.	Test
113 K	5 K	5 K

**Table 3:** Training cost evaluation of vanilla image captioning models. Training eight times for each model.

Model	Train Epoch	Training Time	Params
	(Mean)		
From Scratch	7.125	17 h 52 m	56.13 M
CI (Ours)	6	15 h 24 m	58.23 M

**Table 4:** Metric evaluation of vanilla image captioning models. Training eight times for each model.

Model	BELU-1	BLUE-4	ROUGE-L	METEOR	SPICE	CIDEr-D
	(Mean ± St. Dev.)					
From Scratch	71.78 ± 0.50	29.06 ± 0.31	53.08 ± 0.20	25.58 ± 0.12	19.0 ± 0.06	97.12 ± 0.65
CI (Ours)	72.34 ± 0.27	29.62 ± 0.46	53.46 ± 0.24	26.04 ± 0.16	19.46 ± 0.15	100.24 ± 1.26

**Table 5:** Qualitative analysis of the vanilla image captioning model. The From Scratch model and the CI model were trained while performing inference on the test data image. 1 batch is 1 step, and the batch size is set to 20. 20,705 steps is 1 epoch. *(...repeat...)* means that the previous word is repeated until it reaches the maximum length (180 words).

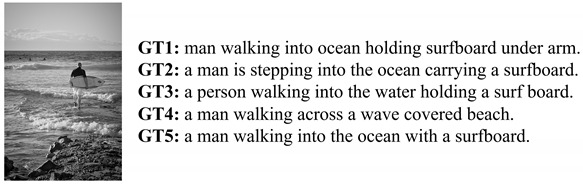		
**Steps**	**Inferences**	
	**From Scratch model**	**CI model**
0	ound congregations mound adverse mound mound congregations mound congregations mound mound *(...repeat...)*	he chapel of the chapel of the chapel was a chapel of the recently to the stag’s stag’s for the <unk>of the <unk>of the <unk>of the <unk><unk><unk>.
200	a a a a a a a a a a	man riding a surf board on a sandy beach.
400	man on a on a on a on a on a on a on a on a on a *(...repeat...)*	man is riding a surfboard on a surfboard.
600	man is is is on a beach.	man is walking on the beach with a surfboard.
2600	man standing on a beach with a surfboard.	man holding a surf board on the ocean.
10,000	man walking on a beach with a surfboard.	man walking on a beach with a surfboard.
20,705	man carrying a surfboard on the beach.	man holding a surfboard on top of a beach.
32,906	man holding a surfboard on top of a beach.	man holding a surfboard standing on a beach.
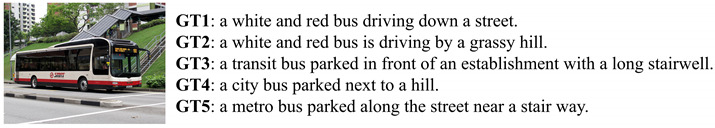		
**Steps**	**Inferences**	
	**From Scratch Model**	**CI Model**
0	slam retreat dilidilidilidilidilidilivietnamese vietnamese vietnamese credits dilicredits dilidil *(...repeat...)*	he <unk>and <unk><unk>were the <unk>of the <unk>and *(...repeat...)*
200	a a a a a a a a a	large double decker bus on a street.
400	is is is on a on a street.	red and white bus on a street.
600	train on a train on a train on a train.	red bus is parked on a street near a building.
1600	bus is parked on a street with a city street.	red and white bus parked on a street.
10,000	bus is parked on the side of a street.	bus is parked on the side of a road.
20,705	bus is parked on the side of the road.	bus is driving down the street near a building.
32,906	red and white bus driving down a street.	bus is stopped at a bus stop.

**Table 6:** Performance comparison according to keyword input percentage. Training eight times for each model.

Input	BELU-1	BLUE-4	ROUGE-L	METEOR	SPICE	CIDEr-D	Train Epoch
(Keywords)	(Mean ± St. Dev.)						(Mean)
30%	72.36 ± 0.29	29.72 ± 0.42	53.44 ± 0.27	26.02 ± 0.15	19.28 ± 0.07	99.80 ± 0.93	6.125
50%	72.73 ± 0.32	29.96 ± 0.27	53.62 ± 0.12	26.04 ± 0.05	19.36 ± 0.17	100.66 ± 0.29	6
70%	72.67 ± 0.14	29.82 ± 0.36	53.62 ± 0.20	26.12 ± 0.16	19.44 ± 0.12	100.90 ± 0.71	6
100%	72.52 ± 0.24	29.76 ± 0.27	53.50 ± 0.25	26.1 ± 0.21	19.36 ± 0.16	100.18 ± 0.96	6.125

**Table 7:** Performance comparison according to FML. *w/o* applies CI without FML. DL 1 is Dense Layer 1, DL 2 is Dense Layer 2, UC is Undercomplete, and OC is Overcomplete. Training seven times for each model.

FML	BELU-1	BLUE-4	ROUGE-L	METEOR	SPICE	CIDEr-D	Train Epoch
	(Mean ± St. Dev.)						(Mean)
*w/o*	71.60 ± 0.15	29.24 ± 0.31	53.06 ± 0.21	25.78 ± 0.12	19.04 ± 0.08	97.86 ± 1.01	6
DL 1	72.26 ± 0.26	29.52 ± 0.25	53.36 ± 0.19	25.82 ± 0.17	19.28 ± 0.10	99.14 ± 0.70	6.71
DL 2	72.40 ± 0.23	29.68 ± 0.17	53.44 ± 0.14	25.98 ± 0.17	19.28 ± 0.13	99.92 ± 0.80	6.71
UC	72.04 ± 0.21	29.48 ± 0.20	53.28 ± 0.12	25.80 ± 0.11	19.10 ± 0.18	98.32 ± 0.19	7.43
OC	72.76 ± 0.40	30.10 ± 0.50	53.66 ± 0.19	26.08 ± 0.10	19.40 ± 0.14	100.96 ± 1.03	6

**Table 8:** Comparison of training cost and parameters of ORT structure for two models, From Scratch and CI. Training seven times for each model.

Model	Train Epoch	Train Time	Params
	(Mean)		
From Scratch	16.4	19 h 24 m	319.6 M
CI (Ours)	12.6	15 h 15 m	320.2 M

**Table 9:** Comparison of metric scores of ORT structure two models, From Scratch and CI. Training seven times for each model.

Model	BELU-1	BLUE-4	ROUGE-L	METEOR	SPICE	CIDEr-D
	(Mean ± St. Dev.)					
From Scratch	76.21 ± 0.26	35.25 ± 0.24	56.16 ± 0.19	27.54 ± 0.12	20.94 ± 0.12	112.96 ± 0.60
CI (Ours)	75.46 ± 0.32	34.56 ± 0.36	55.86 ± 0.17	27.43 ± 0.06	20.69 ± 0.06	111.39 ± 0.81

## Data Availability

Publicly available datasets were analyzed in this study. This data can be found here: Bottom-up-attention image features, https://github.com/peteanderson80/bottom-up-attention (accessed on 7 January 2022); WikiText-103, https://s3.amazonaws.com/research.metamind.io/wikitext/wikitext-103-v1.zip (accessed on 7 January 2022).
